# An Optimal Nucleic Acid Testing Strategy for COVID-19 during the Spring Festival Travel Rush in Mainland China: A Modelling Study

**DOI:** 10.3390/ijerph18041788

**Published:** 2021-02-12

**Authors:** Yu-Hao Zhou, Ke Ma, Peng Xiao, Run-Ze Ye, Lin Zhao, Xiao-Ming Cui, Wu-Chun Cao

**Affiliations:** 1 State Key Laboratory of Pathogen and Biosecurity, Beijing Institute of Microbiology and Epidemiology, Beijing 100071, China; zhouyuhao1994@126.com (Y.-H.Z.); make19920123@163.com (K.M.); xp_4030@163.com (P.X.); runze.ye@mail.sdu.edu.cn (R.-Z.Y.); cuixm7@163.com (X.-M.C.); 2 Institute of EcoHealth, School of Public Health, Cheeloo College of Medicine, Shandong University, Jinan 250012, China; zhaolin1989@sdu.edu.cn

**Keywords:** COVID-19, microsimulation model, SEIR model, testing strategies, public health control

## Abstract

Western countries are experiencing surges in COVID-19 cases and deaths due to increasing public transportation during holiday seasons. This study aimed to explore whether mainland China will face an epidemic rebound during the Spring Festival holiday, when millions of Chinese people travel across the country, and investigate which nucleic acid testing (NAT) strategy is optimal to contain the epidemic. A microsimulation model was used to simulate SARS-CoV-2 transmission among railway travelers and evaluated the effects of various NAT strategies. An extended susceptible-exposed-infectious-recovered (SEIR) model was built to forecast local transmission during the Spring Festival period under different scenarios of testing strategies. The total number of infections, testing burden, and medical expenditure were calculated to devise an optimal strategy during the Spring Festival travel rush. Assuming the daily incidence of 20 per 10 million persons, our model simulated that there would be 97 active infections on the day of travel among 10 million railway passengers without NAT and symptom screening. Pre-travel testing could reduce the number of active infections. Compared with no NAT, testing passengers from risk tier 2–4 regions 3 days before travelling could significantly reduce the risk of transmission, and it is more economical and efficient than testing for all passengers.

## 1. Introduction

Since the first report of the novel coronavirus in Wuhan, China in December 2019, the causative virus SARS-CoV-2 has spread globally, with an unprecedented surge in the number of cases and deaths [1]. To curb the spread of the pandemic, governments around the world have implemented the most stringent control measures, including international and domestic travel restrictions [2]. According to the website FlightAware Real-time Worldwide Flight Traffic [3], global commercial and cargo flights decreased by 80% in June 2020 compared to January, and has since recovered slowly to around 60% in December. Meanwhile, the epidemic curve that had been flattened in June has increased again steeply in recent months [4].

As the holiday season begins, many countries are experiencing surges in COVID-19 cases and deaths. Based on the data from the Transportation Security Administration [5], around one million Americans flew every day during the Thanksgiving holiday (between 20–29 November 2020). After this, COVID-19 cases surged in the first week of December, with the United States reporting record-breaking numbers in cases, hospitalizations, and deaths [4]. At the same time, Germany, the United Kingdom, and Japan set stricter lockdown measures to control a big surge in coronavirus infections.

China has been largely praised for the effective and swift control of the coronavirus outbreak by the World Health Organization [6]. Since December, however, China has faced great threats of cases imported from abroad, and the pressure to detect and control local transmitted cases is increasing (Table S1) [7]. The government has thus urged the public to avoid unnecessary travel, and travelers have been advised to carry a nucleic acid testing (NAT) certificate. The Spring Festival travel rush is a huge-scale phenomenon of high-pressure transportation that occurs around the Lunar New Year in China [8]. A report from the Ministry of Transport of the People’s Republic of China shows that 143 million railway transportation trips were made before the 2019 Spring Festival holiday [9]. Considering the latest situations, the Chinese authorities have raised concerns over a second wave of COVID-19 infections, especially during the upcoming 2021 Spring Festival travel rush.

The objectives of this study include: (1) to explore the efficiency of different nucleic acid testing strategies on railway passengers; (2) to simulate epidemic situations under different scenarios after the Chinese Spring Festival holiday; (3) to answer whether railway passengers will be required to present a negative nucleic acid testing result before travelling; and (4) to devise an optimal nucleic acid testing strategy during the Spring Festival travel rush.

## 2. Materials and Methods

### 2.1. Simulation of the Populations

Populations who potentially travelled by Chinese railways were simulated based on an extended microsimulation model of SARS-CoV-2 transmission [10]. A total of 10 million passengers were estimated to travel by train in mainland China every day during the Spring Festival travel rush. Travelers fall into one of six states of health at a specified time point: susceptible, exposed, pre-symptomatic, symptomatic-infectious, asymptomatic-infectious, and recovered. Parameters of COVID-19 natural history were referred to from the published studies, including the incubation and infectious period, proportion of asymptomatic infection, and pre-symptomatic transmission (Table 1) [11,12,13,14,15,16]. We set the incubation and infectious period as 6 and 5 days, respectively [11,12]. Because the prevalence of asymptomatic SARS-CoV-2 infection has been reported in a wide range between 6.3% to 96% [13,14], we chose the midpoint of 50%. The proportion of transmission during the pre-symptomatic period was set as 50%, according to a previous study [15]. We used published literature on the sensitivity and specificity of PCR assays for SARS-CoV-2, incorporating time-varying estimates of sensitivity based on time since exposure [16]. We set that each individual had a fixed probability of being infected every day, with a slightly increased risk on the day of travel [17,18]. Based on a previous estimation [19], the COVID-19 daily incidence was set at 20 per 10,000,000 persons. The daily incidence varied from 1 to 100 infections per 10,000,000 persons in sensitivity analysis. The simulations ran from 1 December 2020 to 28 January 2021 (the first day of the 2021 Spring Festival travel rush). We continuously counted the number of active infections on the day of travel.

### 2.2. Simulation of Testing Strategies

Once the populations had been simulated, the effects of the six following testing strategies on railway passengers before travelling were evaluated: (a) no NAT required; (b) NAT within 14 days before travelling, i.e., passengers were tested 2–14 days before the travelling day (the standard turnaround time for PCR tests is one day); (c) NAT within 10 days before travelling; (d) NAT within 7 days before travelling; (e) NAT within 5 days before travelling, and (f) NAT within 3 days before travelling. Effects of testing strategies were simulated with or without symptom screening. We assumed that people who were symptomatic or had tested positive for COVID-19 were adhering to self-isolation guidelines and were avoiding travel by train. According to a published study, the sensitivity and specificity of RT-PCR tests were assumed to be 80–95% and 99.5–100% in the first two weeks after exposure, respectively [16,19].

### 2.3. Simulation of Local Transmission during the Spring Festival Period

We used an extended SEIR model to forecast local transmission during the Spring Festival period (Figure S1). The simulation period ranges from Spring Festival travel rush (from 28 January to 10 February 2021) to Spring Festival holiday (from 11 to 17 February 2021). The parameters we used were the same as the microsimulation model (Table 1). The effective reproduction number (*R_t_*) was assumed to fluctuate between 1.25 and 1.75 (Figure S2) and was modified according to the data on population activity of different provinces and municipalities in the same period in 2019 [20]. Based on the daily number of imported and locally transmitted cases since December, the risk of COVID-19 transmission in all 31 provincial-level administrative regions in mainland China were classified into four tiers (Table S1). The number of railway passengers between each province and travel destination was calculated from the 2019 Spring Festival travel rush [9]. Scenarios simulated by the model included: (1) NAT is not required for passengers travelling in all regions; (2) passengers from all regions will be required to undergo NAT for COVID-19 within 7 days before travelling; (3) passengers from all regions will be required to undergo NAT for COVID-19 within 5 days before travelling; (4) passengers from all regions will be required to undergo NAT for COVID-19 within 3 days before travelling; (5) all passengers from high-risk regions (risk tier 3–4) will be required to undergo NAT for COVID-19 within 3 days before travelling; (6) all passengers from medium- and high-risk regions (risk tier 2–4) will be required to undergo NAT for COVID-19 within 3 days before travelling. The number of active infections used in each scenario was calculated from the results of the above microsimulation model.

## 3. Results

### 3.1. Simulated Populations

The microsimulation model predicted that there would be 97 (95% CI: 69, 133) active infections per day among the simulated population during the Spring Festival travel rush in the absence of NAT and symptom screening. Of them, 47 (95% CI: 33, 72) were asymptomatic. On the contrary, if there was symptom screening (still no NAT), the number of daily active infections would be reduced to 72 (95% CI: 51, 110).

### 3.2. Effectiveness of Testing Strategies

**Figure 1:**
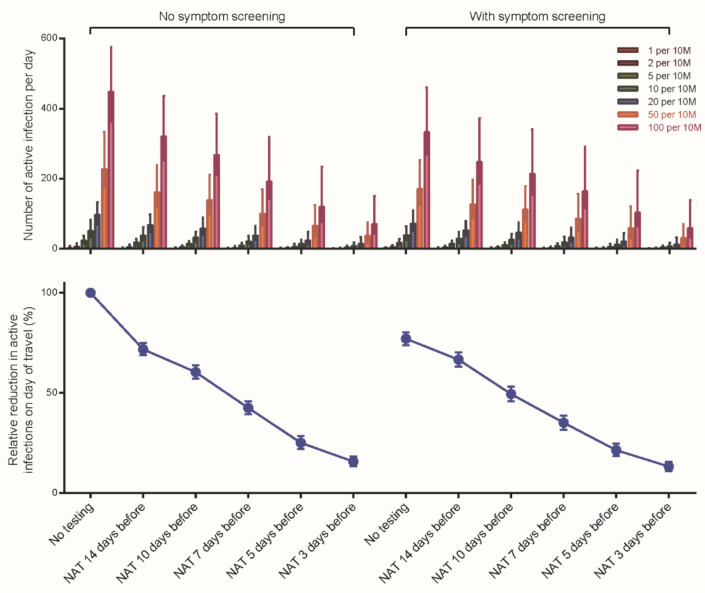
Predicted number of active infections on the day of travel by different testing strategies. We estimated the number of active infections on the day of travel (vertical axis) with simulation of each testing strategy (grouped horizontal axis). The height of the histogram represented the mean and the error bars represented by the 95% confidence interval (95% CI) across 1000 simulations. Each testing strategy was compared under circumstances with or without symptom screening. A sensitivity analysis that was conducted by changing daily incidence varied from 1 to 100 per 10,000,000 persons. Relative reduction in active infections on day of travel by each testing strategy is shown in bottom panel. NAT—nucleic acid testing. 10 M—10 million persons.

We compared the effects of five pre-travel NAT strategies at different time points based on the results of microsimulation model. Compared with the strategies of (a) no NAT, (b) NAT within 14 days before travelling would reduce the number of active infections on the day of travel by 28.1% (95% CI: 25.1, 31.1); (c) NAT within 10 days before travelling would reduce the number by 39.5% (95% CI: 36.2, 42.7); (d) NAT within 7 days before travelling would reduce the number by 57.4% (95% CI: 54.1, 60.7); (e) NAT within 5 days before travelling would reduce the number by 74.8% (95% CI: 71.5, 78.0); and (f) NAT within 3 days before travelling would reduce the number by 84.2% (95% CI: 81.8, 86.7) (Figure 1 and Figure S3).

### 3.3. Local Transmission during the Spring Festival Period

**Figure 2:**
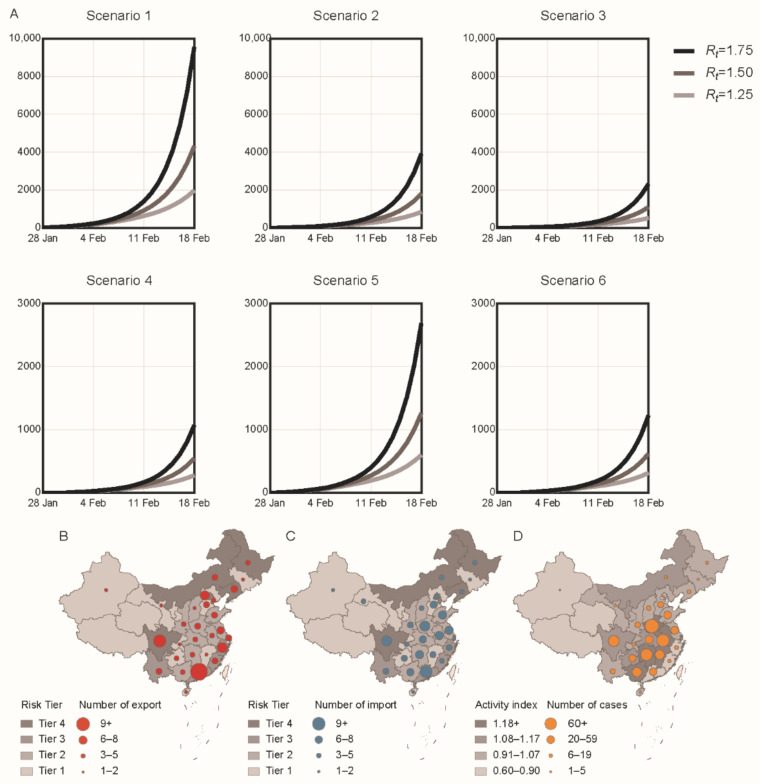
Simulated epidemic in mainland China after the Spring Festival period by different scenarios. (**A**) We forecast the total number of cases (vertical axis) in mainland China after the Spring Festival period by different scenarios (panels). Different grayscale lines represent different values of *R_t_*. (**B**–**D**) Number of exported cases, imported cases, and locally transmitted cases in 31 provincial-level regions by scenario 6. The grayscale of the map represents the risk tier and population activity index in each region. The circles represent the number of cases. *R_t_*—effective reproduction number.

Based on the locally transmitted risk, Beijing, Heilongjiang, Inner Mongolia, Liaoning, Sichuan, and Tianjin were classified as risk tier 4 regions, with relatively high assumed incidence in mainland China. As of 18 February 2021, the total number of infections were estimated according to different values of *R_t_*. In scenario 1, no NAT was conducted for passengers travelling in all regions, and there would be 429 active infections travelling by train. Under these circumstances, the total number of infections would be 1977 (when *R_t_* = 1.25), 4323 (when *R_t_* = 1.5), and 9568 (when *R_t_* = 1.75). In scenario 2, passengers from all regions would have NAT within 7 days before travelling, and there would be 193 active infections. Under these circumstances, the total number of infections would be 840 (when *R_t_* = 1.25), 1804 (when *R_t_* = 1.5), and 3916 (when *R_t_* = 1.75). In scenario 3, passengers from all regions would have NAT within 5 days before travelling, and there would be 127 active infections. Under these circumstances, total number of infections would be reduced to 523 (when *R_t_* = 1.25), 1095 (when *R_t_* = 1.5), and 2316 (when *R_t_* = 1.75). In scenario 4, passengers from all regions would have NAT within 3 days before travelling, and there would be only 81 active infections travelling by train. Under these circumstances, total number of infections would be 278 (when *R_t_* = 1.25), 544 (when *R_t_* = 1.5), and 1072 (when *R_t_* = 1.75). In scenario 5, passengers from high-risk regions would have NAT within 3 days before travelling, and the number of active infections would rebound to 141. Under these circumstances, total number of infections would be 599 (when *R_t_* = 1.25), 1263 (when *R_t_* = 1.5), and 2689 (when *R_t_* = 1.75). In scenario 6, passengers from medium- and high-risk regions would have NAT within 3 days before travelling, and there would be 90 active infections travelling by train. Under these circumstances, total number of infections would be 315 (when *R_t_* = 1.25), 618 (when *R_t_* = 1.5), and 1226 (when *R_t_* = 1.75) (Figure 2A). In scenario 6, Guangdong, Sichuan, Beijing, Zhejiang, and Liaoning were the largest exporters of active infections (Figure 2B). Simultaneously, Guangdong, Henan, Sichuan, Jiangsu, and Anhui were provinces with the largest cases imported (Figure 2C). The simulation results showed that Anhui, Henan, Hunan, Sichuan, and Guangxi were the top five provinces with the largest number of locally transmitted cases after the Spring Festival holiday (Figure 2D).

### 3.4. Nucleic Acid Testing (NAT) Burden and Medical Expenditure

**Figure 3:**
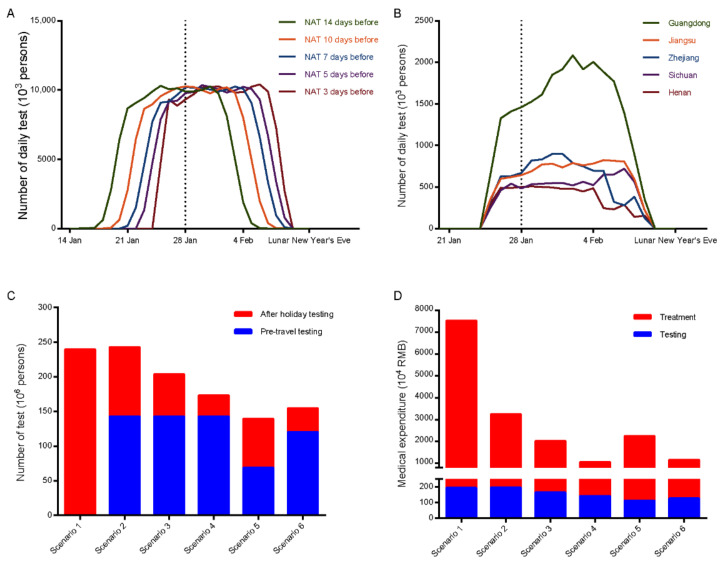
Nucleic acid testing burden of different testing strategies and scenarios. (**A**,**B**) Daily testing amount (vertical axis) of five testing strategies and five provinces with the most demands (if NAT is required 3 days before travelling) was estimated. The vertical dashed line represents the first day of the Spring Festival travel rush. (**C**) Demands of pre-travel testing and after holiday testing in different scenarios. (**D**) The overall medical expenditure in different scenarios. NAT—nucleic acid testing.

We calculated the daily NAT burden of different testing strategies. As shown in Figure 3, the daily testing number of different strategies all peaked around 10 million. The more stringent the testing strategy is, the longer the testing peak lasts, and the more concentrated it is before the Spring Festival holiday. We found that Guangdong, Jiangsu, Zhejiang, Sichuan, and Henan had the heaviest testing burdens, with the highest test demands exceeding 500,000 per day. Among them, Guangdong had the highest testing burden of about 2 million on 2 February 2021.

In scenario 1, 429 active infections were estimated to spread to 30 provinces (except Tibet) by railway transportation with thousands of locally-transmitted cases. In mainland China, community-based NAT was required to contain the outbreak in certain areas. We assumed that the policy of “Testing Everyone Who Should be Tested” would be implemented in each county or district-level region, with more than 10 clustered COVID-19 cases [21]. The total burden of NAT under the six scenarios would reach 239, 242, 203, 173, 139, and 154 million persons after the Spring Festival holiday, respectively (Figure 3C). Including the cost of treatment, the overall medical expenditure would be 75.15, 32.35, 20.00, 10.43, 22.34, and 11.49 million RMB, respectively (Figure 3D).

## 4. Discussion

The COVID-19 pandemic has dramatically changed daily life for people, and it has significantly reduced domestic travel in mainland China [22]. According to data released by the Ministry of Transport of the People’s Republic of China [23], domestic passenger volume by road, railway, and aviation decreased by 45% in 2020. The domestic travel shrinkages in China may be due to the following reasons: (1) the initiative to reduce unnecessary travel by the public, (2) travel restrictions or quarantine rules required by the government or employers, (3) cancellation of nonessential professional, social, and community gatherings that required travel, and (4) other motivations for reducing travel.

However, the Spring Festival is the most important festival for the Chinese people to get together. During the 15-day pre-holiday travel rush period, millions of Chinese people try to rush home from wherever they are, which marks the largest annual human movement in the world [22]. Considering the elevated total passenger throughput during the Thanksgiving holiday in the United States, and the resurge in the number of cases and deaths after the holiday, whether the Spring Festival travel rush will be the amplifier of COVID-19 transmission throughout China is worrying.

The present study demonstrates that routine NAT on railway passengers during the Spring Festival travel rush could reduce the number of active infections on the day of travel and contain local transmission during the Spring Festival period. Additionally, each strategy has its own advantages and disadvantages. The shorter the period of NAT certification before travelling, the better will be the effects of the testing strategy, but the testing burden will be heavier. Considering the fact that the incidences of COVID-19 in mainland China are relatively low, mandatory NAT in all regions would bring a huge waste of medical resources. In our model, requiring all railway passengers from medium to high-risk regions to take NAT within 3 days before travelling could have obvious effects on containing the local transmission.

Our results suggest that Anhui, Henan, Hunan, Sichuan, and Guangxi have a relatively higher risk of local transmission. The possible reason for this is that these provinces are all labor-exporting provinces, with a large number of travelers returning home during the Spring Festival period, and their family reunions could facilitate community transmissions. In contrast, the locally-transmitted risk of metropolises such as Beijing and Shanghai is relatively low because the population activity of these cities during the Spring Festival may be low. Similarly, the return wave after the Spring Festival holiday may increase the risk of local transmission in metropolises.

Based on our simulations, we suggest that: (1) travelers take a private car instead of using public transportation; (2) travelers travel in advance to avoid rush hour; (3) passengers who have to travel by train or other public transportations are advised to carry a negative NAT certificate, especially those from medium to high- risk regions (risk tier 2–4); (4) travelers adhere to safety rules during the Spring Festival period and keep vigilant and cooperative.

This study has some limitations. First, we set a fixed incidence and *R_t_* for the simulations. However, most of the cases reported in China recently were clustered cases rather than sporadic cases, and some regions planned to take more stringent control measures during the Spring Festival travel rush due to the recent outbreak. Therefore, the assumptions of incidence and *R_t_* may be overestimated. Second, there are still some uncertainties concerning the natural history of COVID-19 and transmission heterogeneity that have been simplified and assumed in this model analysis. Third, we assume that the compliance of the public is 100% in our model, which is difficult to achieve in reality. Last but not least, only railway passengers were included in our simulation, while other modes of public transportation were not, so the simulation results might be underestimated.

In this study, we used a microsimulation model to compare the efficiency of different NAT strategies. Simultaneously, the extended SEIR model was used to simulate the trend of the epidemic during the Spring Festival. Combined with analysis of the NAT burden and the overall medical expenditure, we provided an optimal testing strategy on the timing and scale of NAT on railway passengers; this consisted of timely suggestions to ensure the safety of Spring Festival travel and provide a scientific basis for decision-making.

## 5. Conclusions

Routine NAT for COVID-19 on railway passengers could reduce the number of active infections and the risk of local transmission during the Spring Festival period in mainland China. An optimal strategy is that travelers who are from medium- and high-risk regions need to provide a nucleic acid testing result within 3 days before travelling. This study provides guidance for national COVID-19 testing during the Spring Festival holiday, and these testing strategies should be combined with other public health control measures, including social distancing, universal mask wearing, and reduced travel to minimize potential transmission [24].

## Figures and Tables

**Table 1:** Parameters of microsimulation and extended SEIR model.

Parameters	Value	Range	References
Population size	10,000,000		
Incubation period	6 days	1–17 days	[11,12]
Infectious period	5 days		[11]
Proportion of asymptomatic cases	50%	30–70%	[13,14]
Proportion of transmission during pre-symptomatic period	50%		[15]
Daily SARS-CoV-2 infection incidence		1 to 100 infections per 10,000,000	
Relative risk of COVID-19 infection during day of travel	2	1–10	[17,18]
PCR sensitivity		80–95%	[16]
PCR specificity	99.8%	99.5–100%	[16]
Compliance with testing	100%		
Effective reproduction number		1.25–1.75	

## Data Availability

The data presented in this study are available on request from the corresponding author.

## Supplementary Materials

The following are available online at https://www.mdpi.com/1660-4601/18/4/1788/s1, Figure S1: Schematic diagram of the modified SEIR model, Figure S2: Daily cases and estimated effective reproduction number in mainland China in December, Figure S3: Sensitivity analysis of different testing strategies with varied daily incidence, Table S1: Daily imported and local transmitted cases in mainland China since December.
