# Supplementary material for: An Optimal Nucleic Acid Testing Strategy for COVID-19 during the Spring Festival Travel Rush in Mainland China: A Modelling Study
Source: Int J Environ Res Public Health. 2021 Feb 12;18(4):1788. doi: 10.3390/ijerph18041788 (PMC7918574; doi:10.3390/ijerph18041788)
Supplement: Supplementary file 1 [file ijerph-18-01788-s001.pdf]

**Table S1.** Daily imported and local transmitted cases in mainland China since December.

| Province | Imported cases | Local transmitted cases | Risk tier* | Assumed incidence |
|---|---|---|---|---|
| Beijing | 12 | 24 | 4 | 20 per 10 million |
| Heilongjiang | 1 | 15 | 4 | 20 per 10 million |
| Inner Mongolia | 27 | 10 | 4 | 20 per 10 million |
| Liaoning | 2 | 60 | 4 | 20 per 10 million |
| Sichuan | 32 | 13 | 4 | 20 per 10 million |
| Tianjin | 8 | 1 | 4 | 20 per 10 million |
| Fujian | 24 | 0 | 3 | 10 per 10 million |
| Guangdong | 57 | 0 | 3 | 10 per 10 million |
| Shanghai | 184 | 0 | 3 | 10 per 10 million |
| Yunnan | 11 | 0 | 3 | 10 per 10 million |
| Zhejiang | 12 | 0 | 3 | 10 per 10 million |
| Anhui | 1 | 0 | 2 | 5 per 10 million |
| Guangxi | 1 | 0 | 2 | 5 per 10 million |
| Henan | 10 | 0 | 2 | 5 per 10 million |
| Hubei | 1 | 0 | 2 | 5 per 10 million |
| Hunan | 1 | 0 | 2 | 5 per 10 million |
| Jiangsu | 4 | 0 | 2 | 5 per 10 million |
| Shaanxi | 8 | 0 | 2 | 5 per 10 million |
| Shandong | 8 | 0 | 2 | 5 per 10 million |
| Shanxi | 3 | 0 | 2 | 5 per 10 million |
| Chongqing | 0 | 0 | 1 | 2 per 10 million |
| Gansu | 0 | 0 | 1 | 2 per 10 million |
| Guizhou | 0 | 0 | 1 | 2 per 10 million |
| Hainan | 0 | 0 | 1 | 2 per 10 million |
| Hebei | 0 | 0 | 1 | 2 per 10 million |
| Jiangxi | 0 | 0 | 1 | 2 per 10 million |
| Jilin | 0 | 0 | 1 | 2 per 10 million |
| Ningxia | 0 | 0 | 1 | 2 per 10 million |
| Qinghai | 0 | 0 | 1 | 2 per 10 million |
| Tibet | 0 | 0 | 1 | 2 per 10 million |
| Xinjiang | 0 | 0 | 1 | 2 per 10 million |

* Tier 1 includes 11 regions without confirmed cases.
Tier 2 includes 9 regions where imported cases no more than 10.
Tier 3 includes 5 regions where imported cases over 10 but without local transmitted cases.
Tier 4 includes 7 regions with local transmitted cases.
We assumed that daily incidence in regions of Tier 1-4 varied from 2 to 20 infections per 10 million persons.

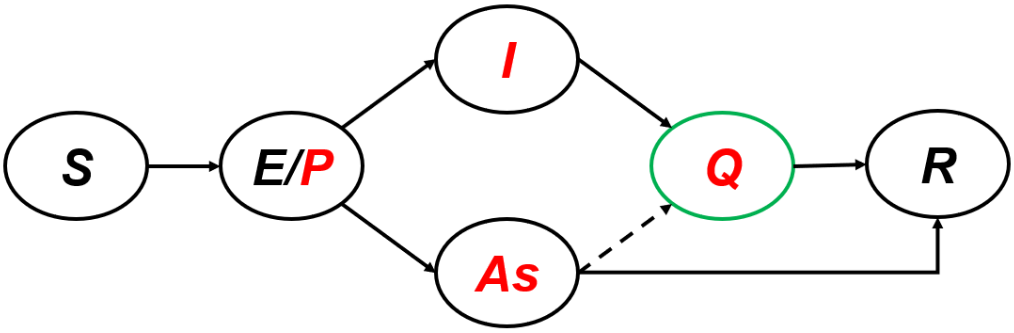


**Figure S1. Schematic diagram of the modified SEIR model.** Travellers fall into one of six states of health at a specified time point: susceptible (S), exposed (E), pre-symptomatic (P), symptomatic-infectious (I), asymptomatic-infectious (As), quarantined (Q) or recovered (R). The red font represents states with infectiousness. The green shield represents individual unable to contact others.

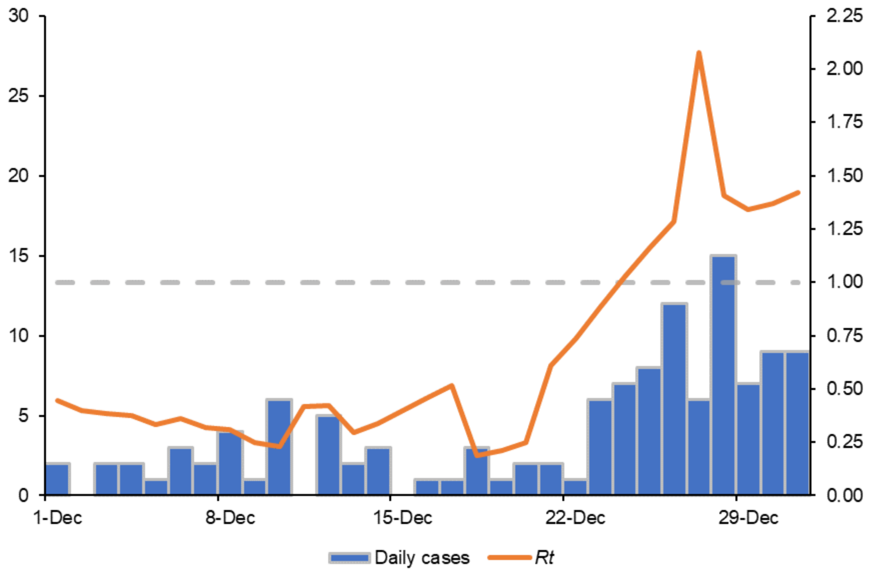


**Figure S2. Daily cases and estimated effective reproduction number in mainland China in December.** The left vertical axis represents daily local transmitted cases. The right vertical axis represents the value of effective reproduction number ($R_t$).

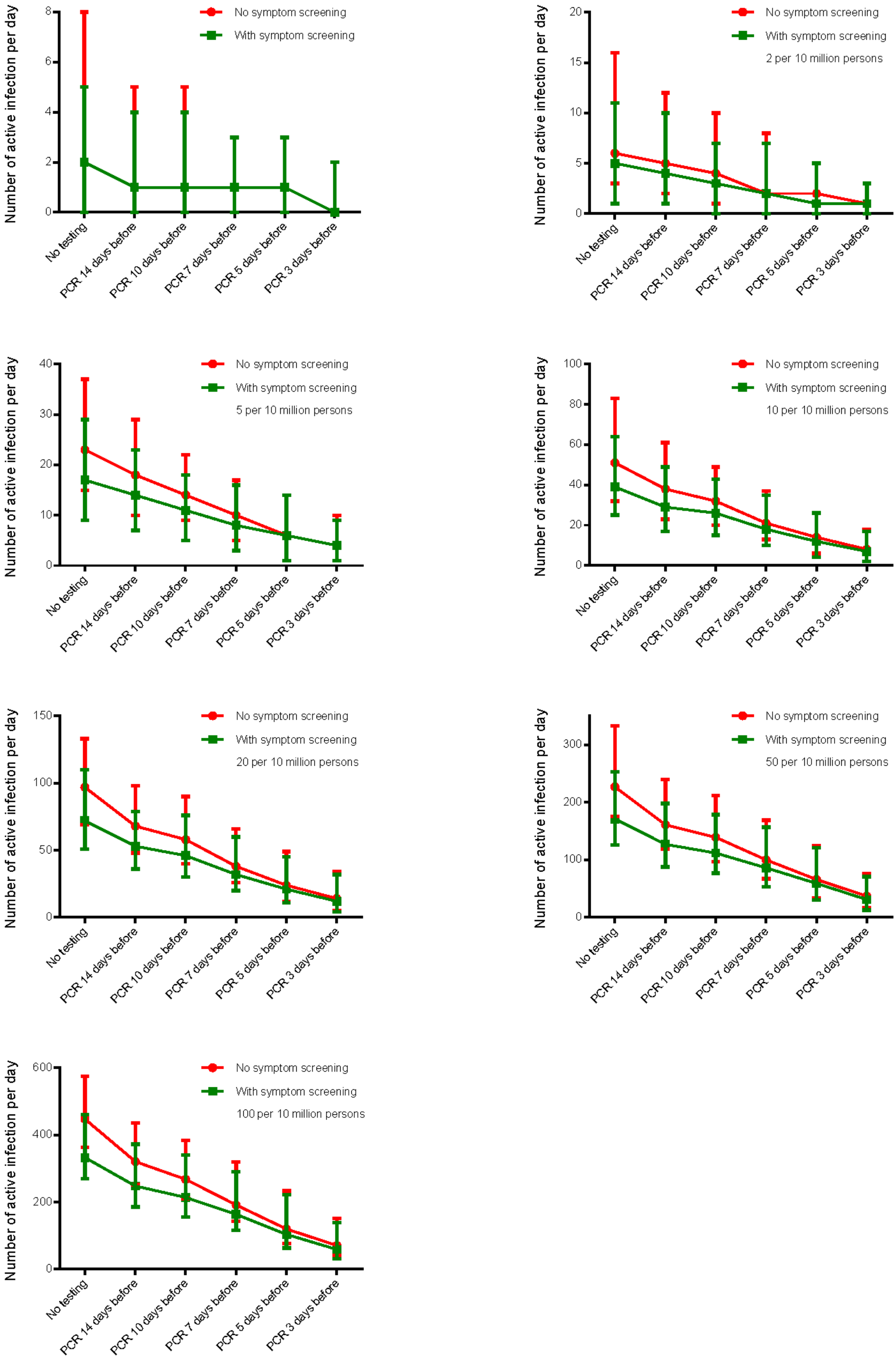


**Figure S3. Sensitivity analysis of different testing strategies with varied daily incidence.** The number of active infections (vertical axis) on the day of travel is estimated with varied daily incidence (panels). The red circle and line represent results of different testing strategies without symptom screening. The green square and line represent results with symptom screening. The error bars represent the 95% confidence interval (95% CI) across 1,000 simulations. NAT, nucleic acid testing.
